# A multicenter retrospective study of neoadjuvant chemotherapy and breast conservation rate in locally advanced breast cancer

**DOI:** 10.1007/s12672-026-04738-2

**Published:** 2026-03-21

**Authors:** Raghda Y. Abu El-Ela, Mohamed Hassan, Rofida Mohamed Kholosy, Loay Kassem

**Affiliations:** 1 Clinical Oncology department, Faculty of medicine, Fayoum University, Fayoum, 63514 Egypt; 2 Clinical Oncology department, Cairo University Hospital, Cairo University, Cairo, Egypt

**Keywords:** Breast cancer, Neoadjuvant chemotherapy, Breast conservation, Pathological complete remission

## Abstract

**Background:**

The burden of locally advanced breast cancer is increasing in low-middle income countries (LMIC) with major impact on the women’s quality of life (QOL). We aimed to assess the impact of neoadjuvant therapy on the rate of conversion of breast surgery from mastectomy to conservation.

**Patients and methods:**

We conducted a retrospective study on files of patients diagnosed with locally advanced breast cancer (clinical stage IIB to IIIC) in 2 academic hospitals between January 2016 and December 2020 who are eligible for neoadjuvant therapy as per tumor size and\or lymph node involvement. The study was approved by the IRB and all patients signed informed consent form.

**Results:**

A total of 103 consecutive patients were eligible with a mean age of 48 years and 26.2% of them younger than 40 years. The majority (87.4%) presented with clinical stage III, 45% were luminal A-like subtype. After neoadjuvant therapy, 100 patients had surgery, among which 47 patients (47%) could attain breast conservation and 35% achieved pathological complete remission (PCR). Breast conservation could be more commonly achieved in patients < 40 years (*p* = 0.05), those with lower clinical stage (*p* < 0.001) and those with higher KI67 (*p* = 0.017). PCR was more common in high KI67 (*p* = 0.016), and HER2-positive tumors (*p* = 0.003). After a relatively short follow-up, the3-year DFS rate for all study group was 65.9% with a superior 3 year DFS rate in patients who had BCS (78% vs. 56.3% in MRM group); *p* = 0.04.

**Conclusion:**

Even in limited resources setting, neoadjuvant therapy has a valuable role in downstaging and breast conservation highlighting its importance as a cornerstone management in high risk non metastatic breast cancer patients. QOL could be an advantage though it was not directly assessed.

**Supplementary Information:**

The online version contains supplementary material available at 10.1007/s12672-026-04738-2.

## Introduction

The burden of locally advanced breast cancer is increasing in low-middle income countries (LMIC) (Ferlay, J et al.) [1]. The problem is even deeper with higher percentage of patients presenting with locally advanced disease in absence of a consistent efficient early detection programs. Younger than 40 females in consideration of 6.6% of whole BC diagnosis, while those under the age of 35 account for 2.4% and those under the age of 30 account for 0.65% (Anders CK, et al.) (Fredholm H, et al.) [2, 3, 10] For instance, a meta-analysis of published data has shown that around two-thirds of Egyptian patients with breast cancer presented with stage III-IV. (Azim et al., JCO global oncology) [4]. In addition, patients present at a younger age compared to their counterparts in the western countries. Locally advanced breast cancer (commonly described to extend between stage IIB and IIIC) has been described as a clear indication for down staging aiming at resectability which typically result in high rates of mastectomy.

The increasing incidence coupled with the advanced presentation have a major impact on the women’s quality of life (QOL). The QOL compromise is driven by 2 major challenges: first, the impact of the cancer therapies, with mastectomy and (neo)adjuvant chemotherapy being the most troublesome interventions and second, the challenge of disease relapse which is more common with locally advanced presentation (Min SY et al. 2010). In younger patients, these burdens can be even larger on the patient herself, on the family and on the healthcare system (Kwan ML et al., 2010) [5].

Improved treatment and earlier discovery have led to continuously declining death rates, particularly among younger females (Ferguson NL et al.) [6].

To deal with this burden, neoadjuvant systemic therapy can help in reducing both the 2 challenges. By applying effective neoadjuvant therapy downstaging of the locally advanced disease could allow for breast conservation. In addition, effective therapies that result in higher PCR rates have been proven to improve the disease free and overall survival. Survival rates for patients with NAC who achieve a (pCR) are higher than those for those who do not(Rastogi P et al. ), (Kuerer HM et al.), ( Guarneri V et al.) [7–9]. Due to the enhanced metastatic potential of HER2-receptor positive and triple-negative cancer, patients with these subtypes may also benefit from early management of distant micro-metastases (Anders CK et al. ) (Wahba HA et al.) [10, 11].

Finally, personalizing the neoadjuvant treatment to the disease subtype (and later, the adjuvant therapy for residual disease) could provide more improvement in the disease outcome. (Sørlie T et al.), (Sørlie T et al.), (Herschkowitz JI et al.)( Huang E et al.) [12–14].

However, applying a personalized neoadjuvant therapy can be challenging in the LMIC with more resources needed for biopsies, pathological exam, tumor localization, and adherence to the full treatment plan. We conducted a multicenter retrospective study to assess the feasibility, and the impact of neoadjuvant therapy on the rate of conversion of breast surgery from mastectomy to conservation.

## Patients and methods

### Study design

This is a retrospective cohort study that reviewed files of consecutive patients diagnosed with locally advanced breast cancer between January 2016 and December 2020 in 2 academic hospitals in Egypt (Cairo University hospital and Fayoum university hospital).

### Eligibility criteria and study endpoints

Eligible patients should have histologically proven breast cancer clinical stage IIB to IIIC who were candidate for neoadjuvant systemic therapy based on their large size tumors and\or lymph nodal involvement. Patients should have available complete data about the clinical stage, ER, PR, HER2 status and should have completed the neoadjuvant chemotherapy and proceeded for surgical management. Patients who has absolute contraindication for CBS were not included the study (e.g.: inflammatory breast cancer, diffuse micro calcifications, multicentric disease, pregnancy at time of diagnosis). The primary endpoint was the rate of breast conservation and secondary endpoints included rate of PCR, clinical response rate, disease free survival (DFS) and overall survival (OS).

### Treatment protocol

All eligible patients were treated with 4 cycles of anthracycline/cyclophosphamide and 4 cycles of paclitaxel with or without anti-HER2. After completion of neoadjuvant treatment, the patients sent for surgical assessment according to response to neoadjuvant treatment, Conservative breast surgery (CBS) (lumpectomy + sentinel LN biopsy/or axillary LNs dissection) done for eligible patients who achieved good response to neoadjuvant treatment. Tumor bed was localized by marker clips inserted under ultrasound guidance done just before starting treatment to identify the tumour bed, if, patient gets such a good response that the lump is no longer clinically palpable. This is to be replaced by wire localization just before surgical management while biopsy of suspicious axillary nodes was done when possible. All measures were done to ensure good assessment of pathological response to and to prevent excessive surgery). Patients who were not eligible for CBS underwent modified radical mastectomy (MRM) (mastectomy + sentinel LN biopsy/or axillary LNs dissection).

Among our study group, most of patients who expressed Her2 positive tumor received trastuzumab (8 mg/kg loading dose then 6 mg/kg maintenance dose cycle every 3 weeks) concomitant with neoadjuvant paclitaxel then continued for one year or started as adjuvant treatment for completed one year.

All hormone receptor positive patients received adjuvant hormonal treatment, premenopausal female were prescribed to received tamoxifen 20 mg PO daily for at least 5 years, zoladex was added for high risk young patients at dose 3.6 mg SC monthly or 10.8 mg SC every3 months, while aromatase inhibitors (mostly femara 2.5 mg PO daily) were prescribed for postmenopausal patients for at least 5 years.

Postoperative radiotherapy PORT was provided to all patients with either doses: 1- standard dose 45-50.4 GY at 1.8- 2 GY per fractions to whole breast +/-boost dose to tumour bed 10–16 GY at 2 GY per fraction or 2- hypofractionated dose 40- 42.5 GY at 2.66 GY per fraction to whole breast.

### Statistical methods

All data were collected, tabulated and statistically analyzed using MedCalc^®^ Statistical Software version 22.003 (MedCalc Software Ltd, Ostend, Belgium). Mean & standard deviation or median & range used for description of continuous variables, categorical variables expressed by number & percentage. Shapiro-Wilk test used for detecting the normality of distribution of continuous variables. Chi-squared test / Fisher’s exact test were calculated to assess the relationship between two categorical variables. Kaplan-Meier method used to perform survival analysis and plot survival curves. Comparison of survival curves assessed by Log-rank test. Logistic regression analysis used to analyze the relationship between one dichotomous dependent variable and one or more independent variables, p value < 0.05 was considered significant. Disease-free survival calculated from the date of start chemotherapy to the date of relapse/death (events) or last FU (censored). Overall survival calculated from the date of diagnosis to the date of death (event) or last FU (censored).

### Ethical issues

The patient’s data were retrieved from patient’s medical records. The protocol study of thesis was submitted to IRB (Institutional Review Board) of Kasr EL-EIini and secured through the expedited pathway. Human ethics declaration in concordance with the Declaration of Helsinki. All measures were taken to protect confidentiality of the patient’s information.

### Funding issues

This work didn’t receive external funding.

## Results

### Patients characteristics

A total of 103 patients with a mean age of 48 years (SD ± 12) were eligible for our study. 27 patients (26.2%) were younger than 40 years old. Regarding the stage, 13 patients (12.6%) were clinically stage IIB, while 90 patients had stage III (73.8%) before starting neoadjuvant therapy. We applied the St Gallen’s 2013 immunohistochemical surrogates for molecular subtypes and ER was positive in 65 patients and negative in 35 patients, representing 65% and 35% of the study group, respectively. PR was positive in 58 patients and negative in 42 patients. Her2 Neu was positive in 35 patients (35%), and negative in 65 patients (65%). Ki67 was documented for only 62 patients with a cutoff 20%, 51 patients (82.3%) had low levels, while 11 patients (17.7%) had high levels. Our patients were stratified into luminal A-like (HR+, Her2-, low KI67 index); luminal B-like (HR+, Her2+, and/or high KI67); Her2 enriched (HR-& Her2+); and TNBC (HR- and Her2), in 47%, 18%, 17%, and 18%, respectively. Table 1 shows the baseline characteristics of the study group.

**Table 1:** Baseline characteristics of the whole study group

Characteristics	Category		Study group(*n* = 103)	
			No	%
Age group	< 40		27	26.2
	≥ 40		76	73.8
Age Mean ± SD	48 12			
Pathology	IDC ILC Others		84 17 2	81.6 16.5 1.9
SBR Grade	2 3		85 18	82.5 17.5
Clinical stage	IIB IIIA IIIB IIIC		13 53 25 12	12.6 51.5 24.3 11.7
ER	+ve -ve N/A		65 35 3	65 35
*PR*	+ve -ve N/A		58 42 3	58 42
HER2	+ve -ve N/A		35 65 3	35 65
K167	Low High N/A		51 11 41	82.3 17.7
Molecular subtypes	Luminal A-like Luminal B-like Her2 enrich TNBC N/A		47 18 17 18 3	47 18 17 18

### Treatment outcomes

All patients started NAC and most of them completed the 8 cycles (93 patients; 90.3%).

Out of 35 HER2-positive patients, only 32 (91.4%) received neoadjuvant trastuzumab and none received dual antiHER2 blockade. After neoadjuvant therapy, 100 patients had radical surgery, with 47 patients (47%) could attain breast conservation and 53 (53%) had to proceed for MRM. A total of 35 patients (35%) achieved pathological complete remission (PCR) in both the breast and axilla. PCR was more common in high KI67 (PCR rate 72.7% versus 31.4% in low Ki67; *p* = 0.016), and HER2-positive tumors (PCR rate 54.3% versus 24.6% in HER2-negative; *p* = 0.003). Table 2 shows correlation of pCR rate with different variables.

Among the patients who could be converted to CBS, patients younger than 40 years had more chance for down staging (CBS rate 63% versus 41.1% in older patients; *p* = 0.05), in addition to those with lower clinical stage (CBS rates of 69.2%, 61.5%, 21.7%, and 8.3% in stages IIB, IIIA, IIIB and IIIC, respectively; *p* < 0.001) and those with higher KI67 (CBS rate of 81.8% versus 39.2% in low KI67; *p* = 0.017). Table 3 shows correlation of CBS rate with different variables.

### Disease free and overall survival

**Fig. 1:**
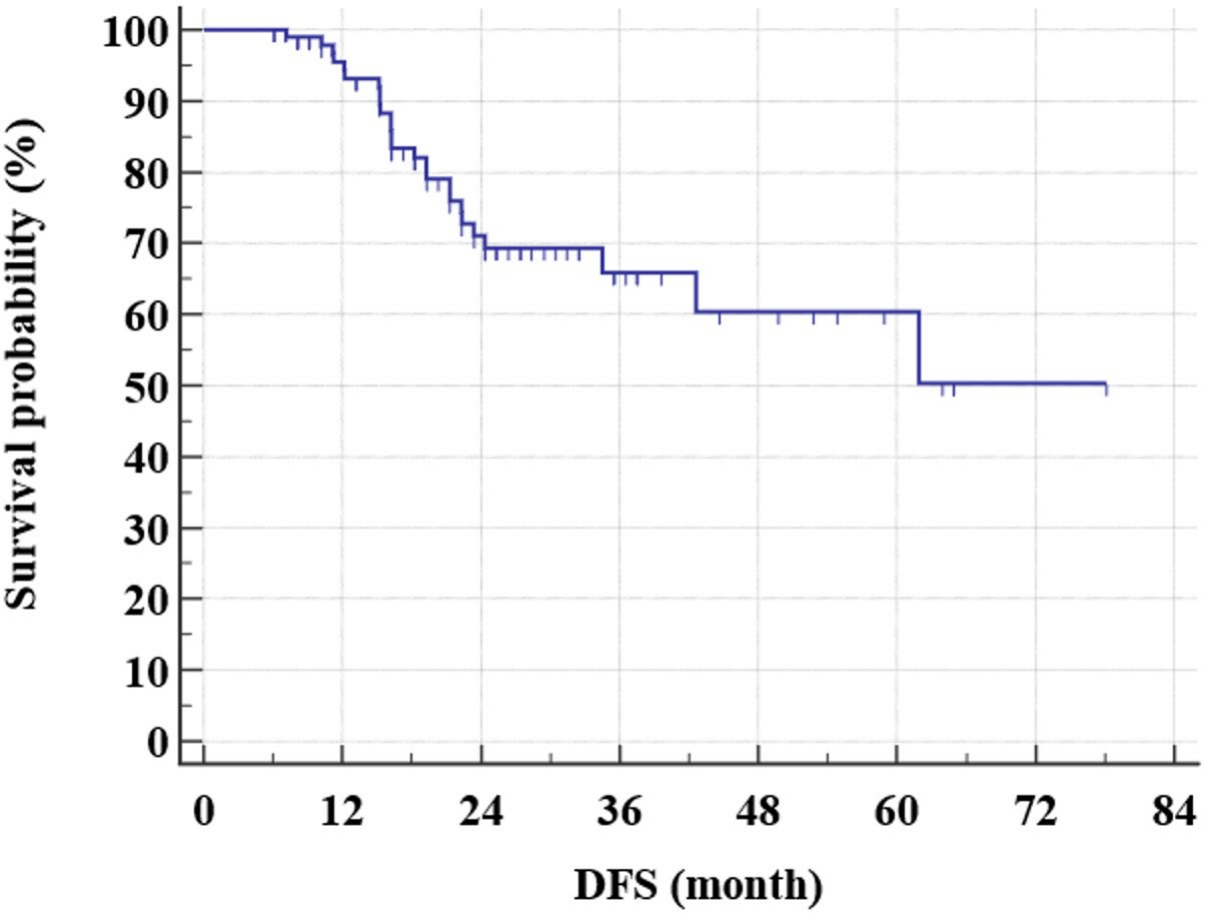
Kaplan-Meier of DFS within the studied patients

**Fig. 2:**
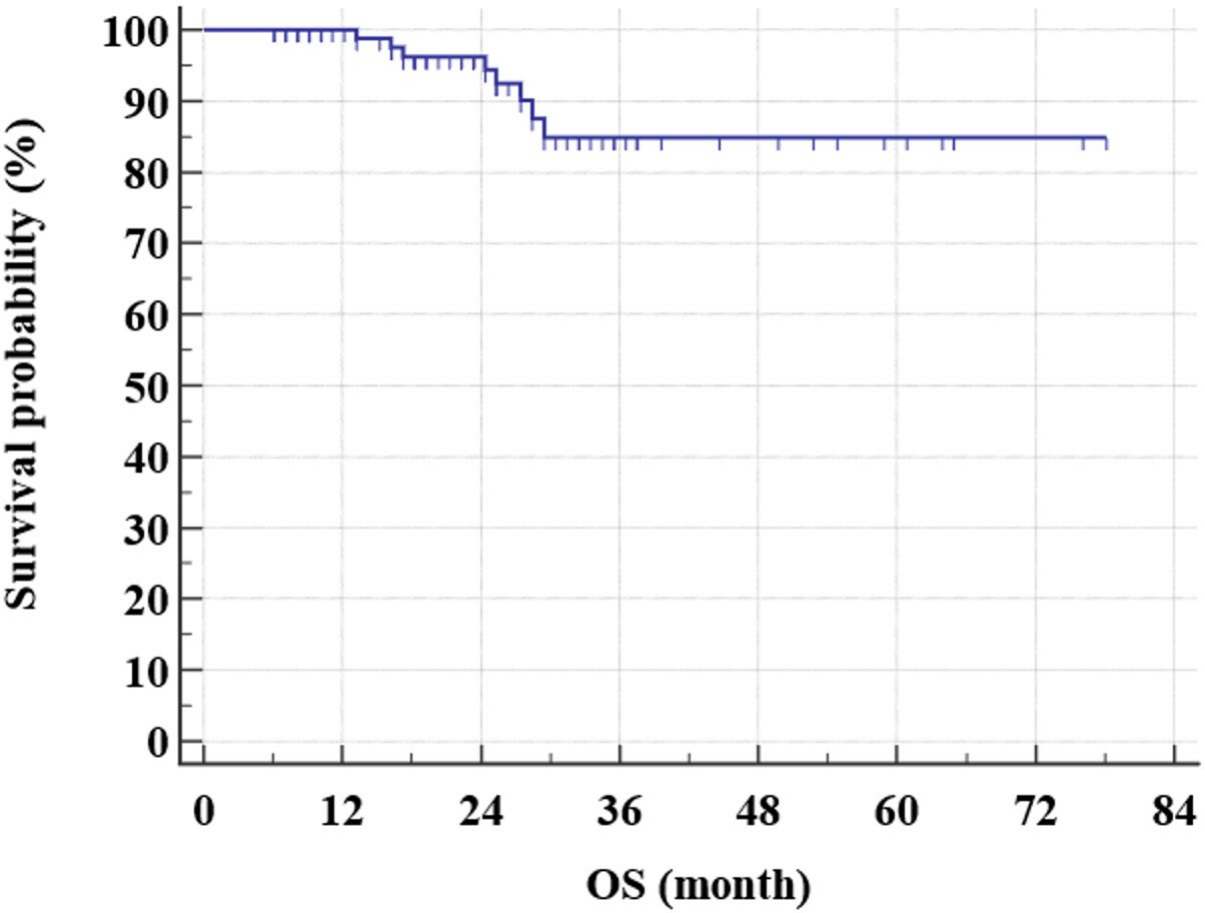
Kaplan-Meier of OS within the studied patients

**Fig. 3:**
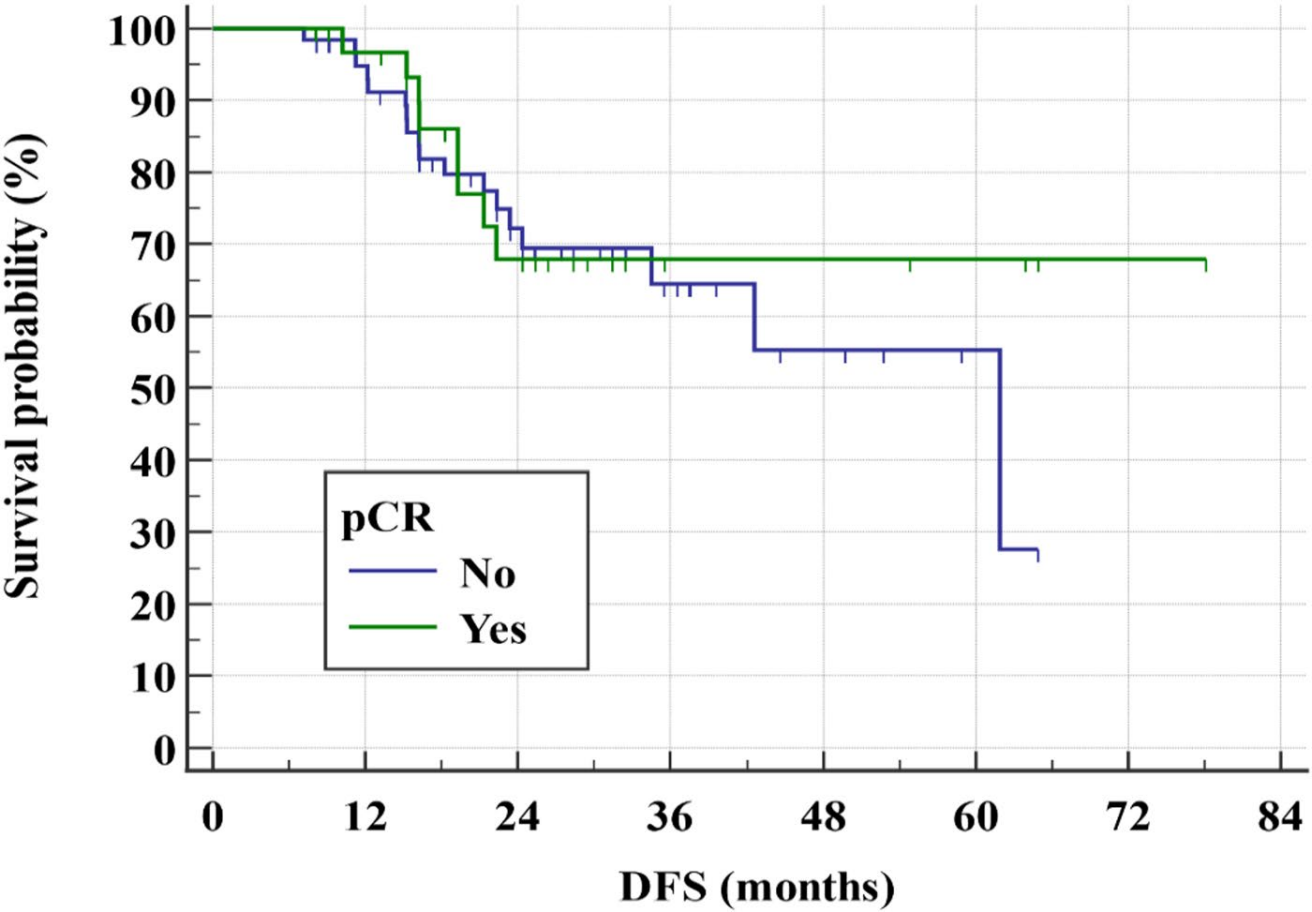
Kaplan-Meier of DFS by the pCR status within the studied patients

**Fig. 4:**
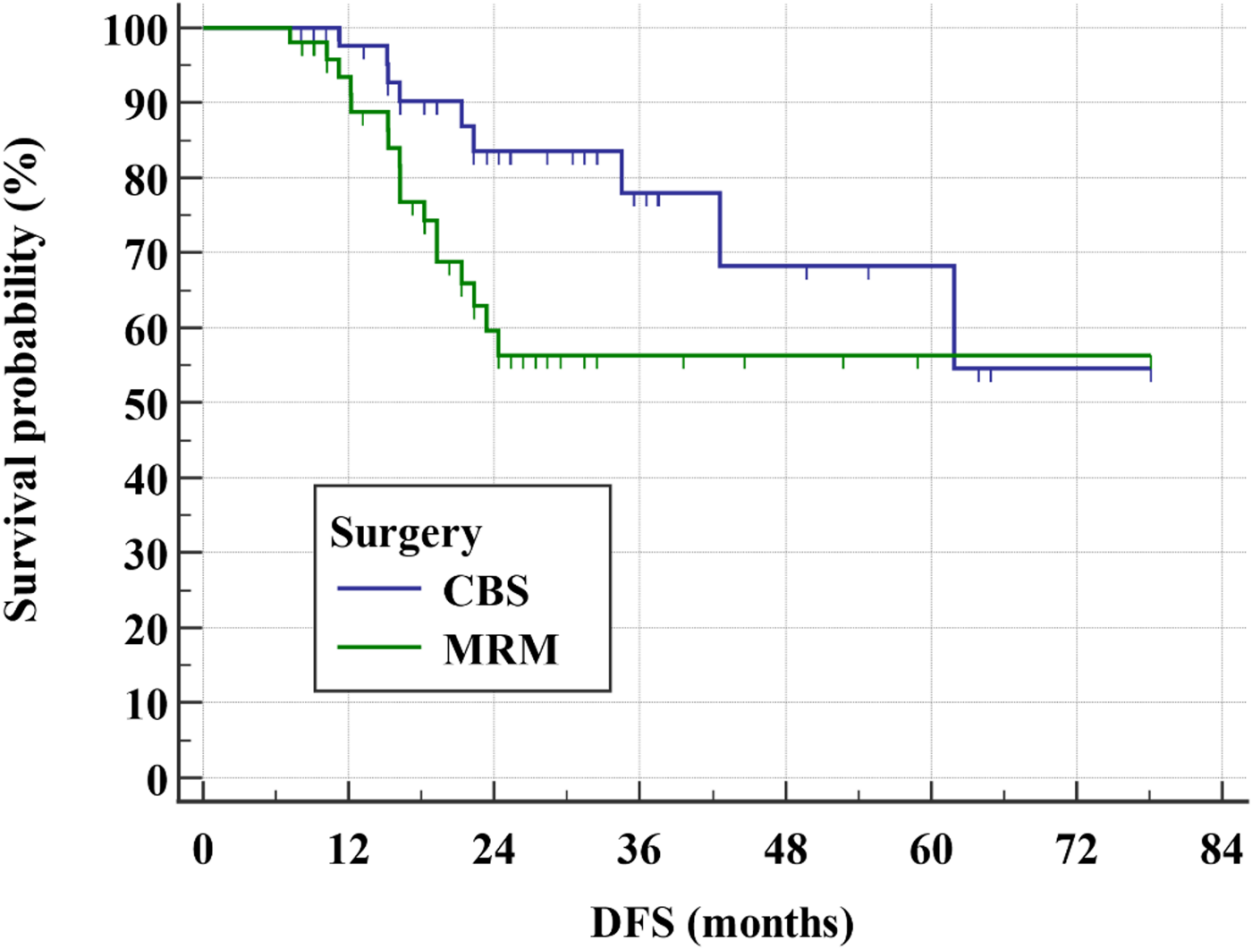
Kaplan-Meier of DFS by the type of surgery within the studied patients

**Fig. 5:**
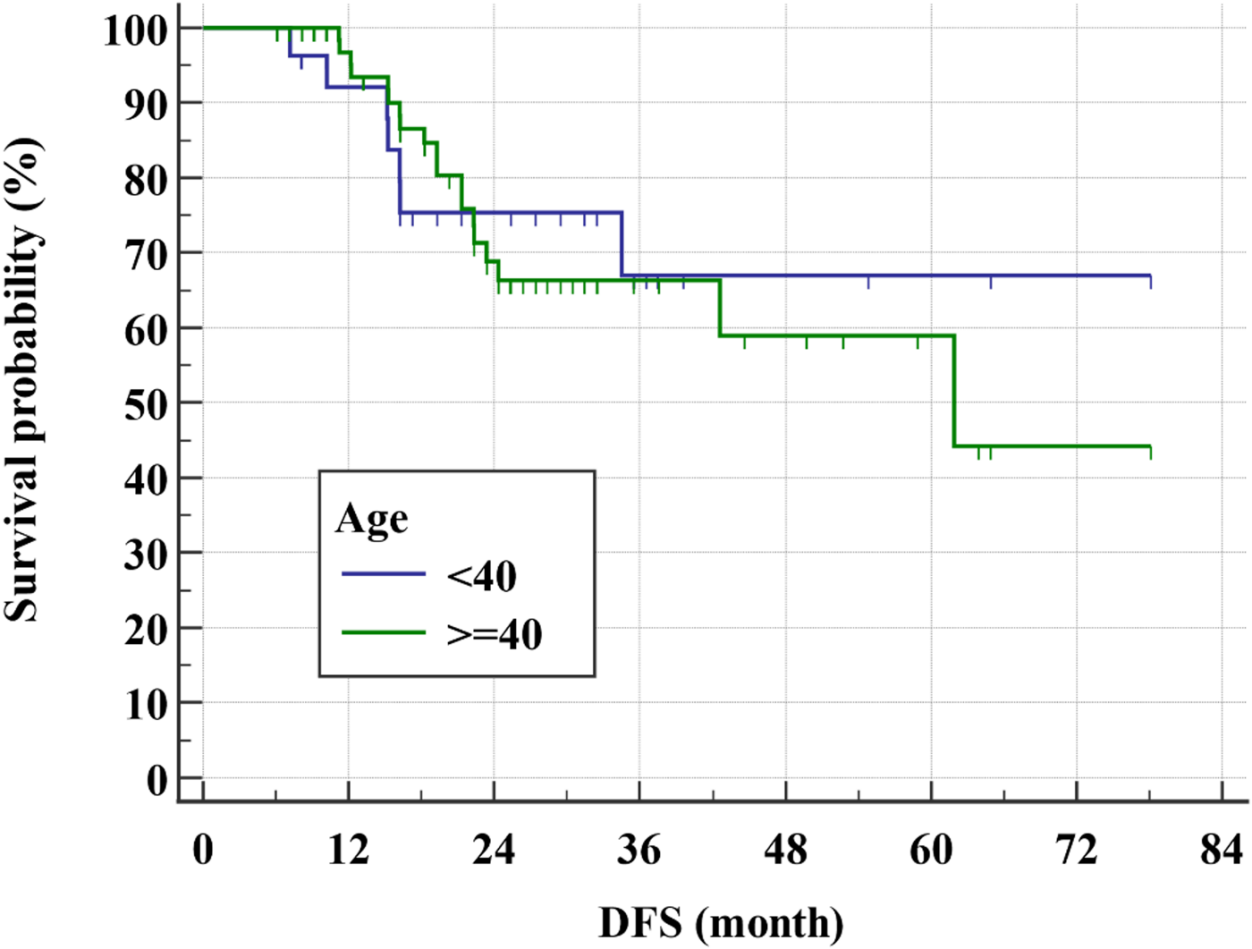
Kaplan-Meier of The association of DFS and age groups within the studied patients

**Fig. 6:**
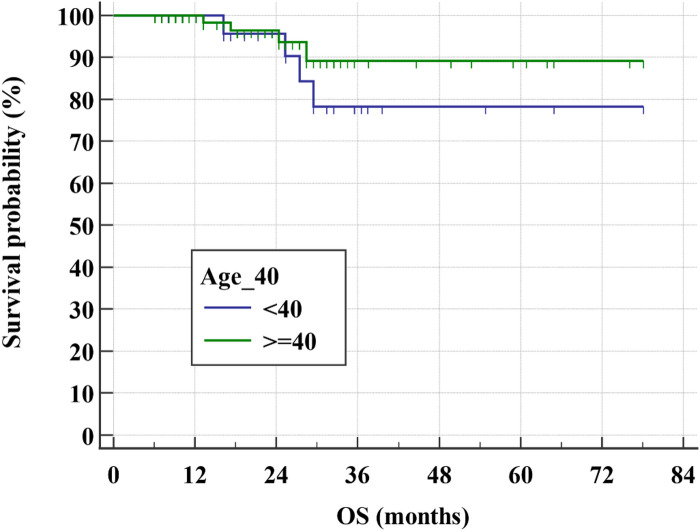
Kaplan-Meier of The association of OS and age groups within the studied patients

After a relatively short follow-up, the 3-year DFS rate for all study group was 65.9% (Fig. 1) while the 3-year OS rate was 84.9% (Figs. 2 and 3). There was even a superior 3-year DFS rate in patients who had BCS with 78% vs. 56.3% in MRM group (*p* = 0.04) (Figs. 4, 5 and 6). Surprisingly, the 3-year DFS rate didn’t show significant difference yet by pCR status with 67.9% in those who achieved pCR versus 64.5% in those with no pCR (*p* = 0.535).

**Table 2:** Correlation of pCR rate with different variables

Variable	Study group				*P* value
	pCR (*n* = 35)		No pCR (*n* = 65)		
	No	%	No	%	
**Age** < 40 ≥ 40	13 22	48.1 30.1	14 51	51.9 69.9	0.0953
**Clinical stage** IIB IIIA IIIB IIIC	5 20 5 5	38.5 38.5 21.7 41.7	8 32 18 7	61.5 61.5 78.3 58.3	0.5021
**ER** +ve -ve	22 13	33.8 37.1	43 22	66.2 62.9	0.7429
**PR** +ve -ve	19 16	32.8 38.1	39 26	67.2 61.9	0.5827
**Her2** +ve -ve	19 16	54.3 24.6	16 49	45.7 75.4	**0.0032**
**Ki67** Low High	16 8	31.4 72.7	35 3	68.6 27.3	**0.0165**
**Molecular subtypes** Luminal A Luminal B Her2 enrich TNBC	12 10 9 4	25.5 55.6 52.9 22.2	35 8 8 14	74.5 44.4 47.1 77.8	**0.0308**

**Table 3:** Correlation of CBS rate with different variables

Variable	Study group				*P* value
	CBS (*n* = 47)		MRM (*n* = 53)		
	N0	%	NO	%	
**Age** < 40 ≥ 40	17 30	63 41.1	10 43	37 58.9	0.0529
**Clinical stage** IIB IIIA IIIB IIIC	9 32 5 1	69.2 61.5 21.7 8.3	4 20 18 11	30.8 38.5 78.3 91.7	**0.0002**
**ER** +ve -ve	30 17	46.2 48.6	35 18	53.8 51.4	0.8182
**PR** +ve -ve	28 19	48.3 45.2	30 23	51.7 54.8	0.7650
**Her2** +ve -ve	18 29	51.4 44.6	17 36	48.6 55.4	0.5171
**Ki67** Low High	20 9	39.2 81.8	31 2	60.8 18.2	**0.01743**
**Molecular subtypes)** Luminal A Luminal B Her2 enrich TNBC	19 11 7 10	40.4 61.1 41.2 55.6	28 7 10 8	59.6 38.9 58.8 44.4	0.3894

## Discussion

We conducted a retrospective study on locally advanced breast cancer who were only candidates for mastectomy. We could show that with successful chemotherapy, downstaging of breast cancer and having breast conservation was feasible and safe in this cohort of Egyptian, young patients despite using relatively older neoadjuvant systemic therapy regimens. This is the first study that is dedicated to answer such question in Egyptian population with findings that are reassuring for all oncologists dealing with such a high-burden disease.

Despite the locally advanced stage in our population, one in every two patients could go for CBS, and 35% could achieve pCR. This denotes the value of neoadjuvant therapy and the needed awareness of multidisciplinary teams of such results when counselling breast cancer patients. Being able to attain breast conservation can have an expected good impact on patients’ psychology and quality of life (QOL) (Kim et al.) [15]. This point is of even more importance in Egyptian population, which was found to be at least 10 years younger than the western patients as described in an analysis of the SEER database Schlichting et al.) [16].

Many studies indicate the role of NACT in down staging and breast conservation in initially inoperable breast cancer. In a study by Oriana Petruolo et al. [17], where 600 of 1353 cancers were CBS-ineligible with large tumors; 69% were non-BCS candidates; and 31% were borderline-CBS candidates. Of non-BCS candidates, 69% became CBS-eligible after the NACT and among the borderline-CBS candidates, 87% were BCS-eligible after NACT. Our study concludes similar findings even in a higher stage population.

Surprisingly, in our study, DFS in patients who had CBS was superior compared to those who had MRM with (*p* = 0.04). This is consistent with data revealed by Simons et al. [18], which estimated survival rates in CBS vs. MRM after NACT, where DFS was 90.9% for CBS versus 82.9% for MRM (*p* = 0.004). This result might be of course, confounded by the fact that patients who went for mastectomy were those with more advanced tumours and poor response to neoadjuvant chemotherapy.

The pCR rate of 35% represents an encouraging result while we acknowledge that the neoadjuvant regimen used was sub-optimal with the 2025 standards. Most of our patients (90.3%) received a sequential anthracycline followed by taxanes regimen. While this regimen might be enough for ER+/HER2- patients, it is not adequate for more aggressive subtypes like TNBC and HER2 + tumours. Her2 + ve and TNBC patients represented 17% and 18% of the study population, respectively. The standard neoadjuvant therapy for HER2 + tumors entail the addition of dual HER2 blockade with trastuzumab + pertuzumab. In the NeoSphere study, patients treated with neoadjuvant pertuzumab, trastuzumab, and docetaxel had a significantly higher pCR rate compared with those treated with trastuzumab plus docetaxel only (45.8% versus 29.0%, respectively). Gianni et al. [19]. In our series, the pCR was higher in her2 + ve patients (54.3%) compared to HER2-ve patients (24.6%) (*p* = 0.0032). No patients in our series received dual anti-HER2 blockade and 91.4% of the HER2 + ve patients received neoadjuvant trastuzumab concurrent with taxanes and continued adjuvant treatment for a total of one year. Similarly, in TNBC patients, the neoadjuvant regimen didn’t include neither carboplatin nor pembrolizumab. Of note, three randomized studies could prove that the addition of carboplatin to ananthracycline-taxane-based chemotherapy was able to improve the pCR rate and DFS (Geyer. et al. [20]. Recently, the KEYNOTE-522 study could show that adding pembrolizumab to neoadjuvant carboplatin-containing chemotherapy could improve the pCR rates, DFS and OS (Schmid et al. [21].

The high proportion of luminal patients achieving pathologic complete response (pCR), compared with the relatively lower response rates in HER2-positive and triple-negative subtypes, is striking and not typically reported in the literature. We think may be this is due presence of larger number of luminal patients in the study group which is accepted as luminal tumours are already the commonest type in breast cancer that represent around 60% of all breast cancer cases. Also, the kind of systemic treatment, as mentioned before, was not adequate for TN and HER2-positive patients. In addition to multi biological factors affecting response including initial tumour size, grade, proliferation index and tumour necrosis. Also, the fact than TNBC are heterogeneous group with different responses to treatment (e.g.: BL1 subtype has a higher response rate while BL2 and LAR subtypes have lower pCR rates). Finally the presence of tumour associated lymphocytes indicates better response Hu et al. [22].

Our study results are limited by the relatively small sample size, the retrospective nature of the study, and the heterogeneity of the study populations. Also, short term follow up (3 years) has its implications on long term outcome assessment (recurrence rate and overall survival). Taking also in consideration that luminal tumours (being the biggest number of the study group) can have a long time till disease recurrence.

In conclusion, our study could show that even with a sub-optimal neoadjuvant treatment, we could achieve adequate down staging with neoadjuvant chemotherapy resulting in a high rate of breast conservation that would expect to affect QOL (we couldn’t do accurate assessment of QOL within the provided retrospective data) but we would expect its psychological and social impact. In addition to the proven value of achieving PCR with its impact on DFS and OS, our study highlights the importance of wide use of the neoadjuvant strategy in high-risk non-metastatic breast cancer, even in a limited resource setting.

### Recommendations

Despite the sub-optimal neoadjuvant treatment for some patients, we observed an adequate response to neoadjuvant treatment, with 35% of patients achieving PCR, and in addition, despite high clinical staging, 47% of our patients could go for CBS. Conducting a prospective larger cohort study with a modern more personalized neoadjuvant therapy is needed to further confirm our results.

Some of personalized strategies are: addition of dual HER2 blockade with trastuzumab + pertuzumab and docetaxel to achieve a higher PCR rate, addition of carboplatin and pembrolizumab to taxenes in neoadjuvant treatment of stages II and III TNBC to also increase PCR rates and adjuvant capecitabine is recommended in TNBC patients who had residual disease after NACT. The provision of personalized recommended treatment according to the stage and biology is highly recommended.

Finally, we emphasize the importance of a multidisciplinary team including clinical oncologists, pathologists, radiologists, and psychologists to deliver comprehensive care that addresses as many of the patient’s needs as possible.

## Supplementary Information

Below is the link to the electronic supplementary material.

Supplementary Material 1

## Data Availability

All data generated or analysed during this study are included in this published article [and its supplementary information files].

## Abbreviations

BC: Breast cancer
QOL: Quality of life
NAC: Neo-adjuvant chemotherapy
LMIC: Low middle income countries
Pcr: Pathological complete response
Her-2neu: Human epidermal growth factor 2
MRM: Modified radical mastectomy
BCS: Breast conservative surgery
DFS: Disease freee survival
ER: Estrogen receptor
PR: Progesterone receptor
OS: Overall survival

## Declarations

All methods were carried out in accordance with relevant guidelines and regulations.
